# Recombinant Human Epidermal Growth Factor Alleviates Gastric Antral Ulcer Induced by Naproxen: A Non-steroidal Anti Inflammatory Drug

**DOI:** 10.4021/gr2010.05.199w

**Published:** 2010-05-20

**Authors:** Ashok Raja Chairmandurai, Srinivas Vellimedu Kanappa, Krishna Mohan Vadrevu, Uday Kumar Putcha, Vijayalakshmi Venkatesan

**Affiliations:** a Bharat Biotech International Limited, Genome Valley, Shameerpet Mandal, Hyderabad 500 078, Andhra Pradesh, India; b Department of Pathology, National Institute of Nutrition, Jamai Osmania, Tarnaka, Hyderabad-500 007, India; c Department of Biochemistry, Stem Cell Research Laboratory, National Institute of Nutrition, Jamai Osmania, Tarnaka, Hyderabad-500 007, India

**Keywords:** rhEGF, Naproxen, NSAIDs, Gastric-antral ulcer, Healing, WNIN rats

## Abstract

**Background:**

To study the effect (s) of recombinant human Epidermal Growth Factor (rhEGF) on naproxen induced gastric ulcer in Wistar NIN rats.

**Methods:**

Male Wistar NIN rats were randomly divided into six experimental groups: Group I - Control, Group II - Naproxen treated, Group III **-** Naproxen with rhEGF/7 days, Group IV - Naproxen without rhEGF/7 days, Group V - Naproxen with rhEGF/14 days, and Group VI - Naproxen without rhEGF/14 days. Gastric ulcer was induced with naproxen at a concentration of 80 mg/kg by oral administration. After 24 hours of induction of ulcer, rhEGF treatment was started at a concentration of 100 µg/kg. Ulcer presence and healing were confirmed by histopathology study and molecular markers.

**Results:**

Naproxen per se induced gastric antral ulcers in Wistar NIN rats. Compared with control rats, naproxen induced rats had increased lipid peroxide content in serum. Subsequent decrease in lipid peroxide was observed in rhEGF treated groups. Treatment with rhEGF significantly resulted in healing of the ulcers, which was evident by 7 days of rhEGF treatment with total healing seen by 14 days. Significant increase in immunoreactivity for Cox-2 was observed when compared to control groups, whereas less immunoreactivity of Cox-2 was observed in rhEGF treated group. Compared with control group, naproxen induced group exhibited more gene expression of both Cox-2 and TGF beta while gene expression of Cox-2 and TGF beta in rhEGF group was comparable to control group.

**Conclusions:**

The beneficial effects of rhEGF in the management of ulcer healing can be understood. Oral rhEGF can promote healing of the rats with gastric ulcer by stimulating Cox-2 and TGF-beta expression.

## Introduction

Ulcer is a debilitating disease affecting a significant proportion of the population globally [1]. Epidemiological studies show that males have about three times as many ulcers as females between the ages of fifty-five and seventy years. The etiologies of this condition are multifactorial which include *Helicobacter pylori* infection, NSAID toxicity, Zollinger-Ellison syndrome, gastrinoma, smoking and so on. Studies have shown that blood type ‘A’ is more likely to have gastric ulcers, while those with type ‘O’ are more likely to develop duodenal ulcers. To some extent, mental stress also plays an important role in inducing ulcers1.Ulcer healing is a complex process, which involves cell migration, proliferation, reepithelization, angiogenesis, and matrix deposition ultimately, leading to scar formation [2]. Studies in model system have helped to elucidate the pathophysiology and pathobiology underlying the gastric ulcers induced by several factors such as aspirin, alcohol, acetic acid and so on [3]. Ulcer causes inflammatory injuries in the gastric or duodenal mucosa with extension beyond the sub mucosa into the muscularis mucosa leading to imbalance between the gastro-duodenal mucosal defense mechanisms [4].

Naproxen is a non-corticosteroid drug with anti-inflammatory, antipyretic and pain relieving properties used more frequently than other NSAIDs for arthritic patients [5, 6]. However, the major cause of concern with the long term use of naproxen is the development of the gastric antral ulcers [7, 8]. New insights from basic and clinical research suggest that preservation and/or cytoprotection of gastric mucosa is of utmost importance towards the control and management of peptic ulcers. In light of these findings, it appears logical to explore for the promising molecule(s) which may serve as a beneficial tool to develop prophylactic and therapeutic remedies given the exorbitant cost of treating ulcers on the long term basis. Studies by Kim et al [9] have shown the beneficial effects of the astaxanthin - a plant derived extract which was effective in overcoming the naproxen induced gastric antral damage. Similarly, the cytoprotective effects of the growth factor such as Epidermal Growth factor (EGF) were effective towards the restoration of lesioned gastro intestinal mucosal integrity both in vivo and in vitro [10]. In line with these reports it has been demonstrated that orally administered EGF ameliorated the gastric and duodenal mucosa injury induced with the ethanol treatment [11].The Recombinant Human EGF (rhEGF) developed indigenously by Bharat Biotech International Limited has been from expression of a synthetic gene in Escherichia coli similar to the natural EGF with its purity to be 99% assessed by HPLC [12]. Further, its healing functions have been unequivocally demonstrated in diabetic foot ulcers, burns and skin grafts in the human subjects [13].

In light of these findings, it appeared logical for us to explore the beneficial effects of rhEGF against the gastric antral lesions induced with the naproxen treatment on the long term basis using biochemical, cellular and molecular approaches. Further, there are no reports to document the EGF effects on the naproxen induced gastric antral ulcers despite the fact that the gastric antrum forms the primary site of exposure. Further, understanding the healing and repair process with rhEGF also suggests its therapeutic efficacy in patients being treated with long term anti-inflammatory drugs such as NSAID.

## Materials and Methods

### Animals

All animal experimental procedures were approved by the Institutional Ethical Committee on animal research and the institutional guidelines were followed for the care and use of laboratory animals. Six to eight weeks male WNIN rats were obtained from the inbred colony at National Center for Laboratory Animal Sciences, Hyderabad, India. All animals were in a pathogen free environment maintained at 12 hours dark and light cycles with temperature and humidity control. The animals had free access to standard feed and water.

### Experimental design

Thirty-six male WNIN rats were randomly divided into six experimental groups as given in Table 1. Naproxen treated animals received a single dose of 80 mg/kg body weight dissolved in double distilled water administered by orogastric gavage [14]. The animals were sacrificed after 24 hours (Group II), 7 days (Group III) or 14 days (Group V). The rhEGF was administered orally at the concentration of 100 µg/kg body weight [14], to the 24 hours naproxen treated animals for a period of either 7 days (Group IV) or 14 days (Group VI). The Group I animals which received only double distilled water served as the controls. At the end of the experimental duration, the animals were killed by CO_2_ asphyxiation. Stomach was opened along the greater curvature, then rinsed with 0.9% saline, and the gastric antral regions were dissected out and taken for analyses.

**Table 1:** Experimental Design

Group	No. of Animals	Treatment	Duration	Sacrifice After
I	6	Distilled water	1 day	1 day
II	6	Naproxen	1 day	1 day
III	6	Group II + without rhEGF	7 days	7 days
IV	6	Group II + with rhEGF	7 days	7 days
V	6	Group II + without rhEGF	14 days	14 days
VI	6	Group II + with rhEGF	14 days	14 days

All the experimental conditions are as described in materials and methods. Six animals per group have been taken for this study.

### Histopathology

The gastro-antral regions were excised from all the animals, preserved in 10% neutral buffered formalin, and embedded in paraffin, of which 4 µm thick sections were obtained using microtome (Leica RM 3040) and were subsequently stained with Hematoxylin and Eosin (H and E) by the standard method [15].

### Measurement of serum thiobarbituric acid reactive species (TBARS)

Lipid peroxidation was estimated by measuring the thiobarbituric acid reactive species (TBARS) using 1,1,3,3-Tetramethoxypropane [16]. Briefly, 80 μl of serum from the control and experimental groups were mixed thoroughly with freshly prepared thiobarbituric acid and heated at 90°C for 30 minutes in a water bath, cooled to room temperature and centrifuged. The pink colored product was measured at 532 nm for TBARS and the MDA content expressed as nanomoles per litre of serum.

### Immunolocalization of Cox-2

The paraffin sections of 4 µm thickness were mounted onto poly-L-lysine coated slides and were dried overnight at 37°C. Immunohistochemical analysis was carried out using the primary polyclonal antiserum (Goat polyclonal anti Cox-2, 1:250, Santa Cruz, USA), and secondary antibody (FITC labeled Donkey anti goat - 1:500, Molecular probes, U.S.A) [17]. Images were captured on Nikon Eclipse microscope (TE 2000-S series) and the fluorescence intensity units (FIU) were corrected using appropriate controls (Primary antibody controls). The FIU have been quantitated as relative florescence units (RFU) and the experiments have been carried out independently in three sets of experiments.

### Semi-quantitative RT-PCR

Total RNA was extracted from gastro-antral regions by in situ lysis of the tissues with trizol reagent. The integrity of RNA was confirmed by agarose gel electrophoresis (1%). cDNA was prepared from 1.5 μg of total RNA using enhanced avian myeloblastosis virus reverse transcriptase (eAMV-RT) enzyme (Sigma, St. Louis, MO, USA) and the cDNA was amplified with JumpStart AccuTaq LA DNA Polymerase kit (Sigma, St. Louis, MO, USA) according to the manufacturers protocol for genes Cox-2, TGF-β and β actin. PCR products were separated in 1% agarose gel and stained with ethidium bromide. The band intensity was measured using the gel-documentation system (BIO-RAD U.S.A) and quantitated using 1-D analysis software (BIO-RAD U.S.A). Levels of mRNA expression were normalized with those of internal control β actin.

### Statistical analysis

All data have been expressed as means ± SE. One-way analysis of variance (ANOVA) followed by posthoc LSD test with SPSS software was applied to determine the significance. P value less than 0.05 was considered statistically significant.

## Results

### Histopathology

**Figure 1:**
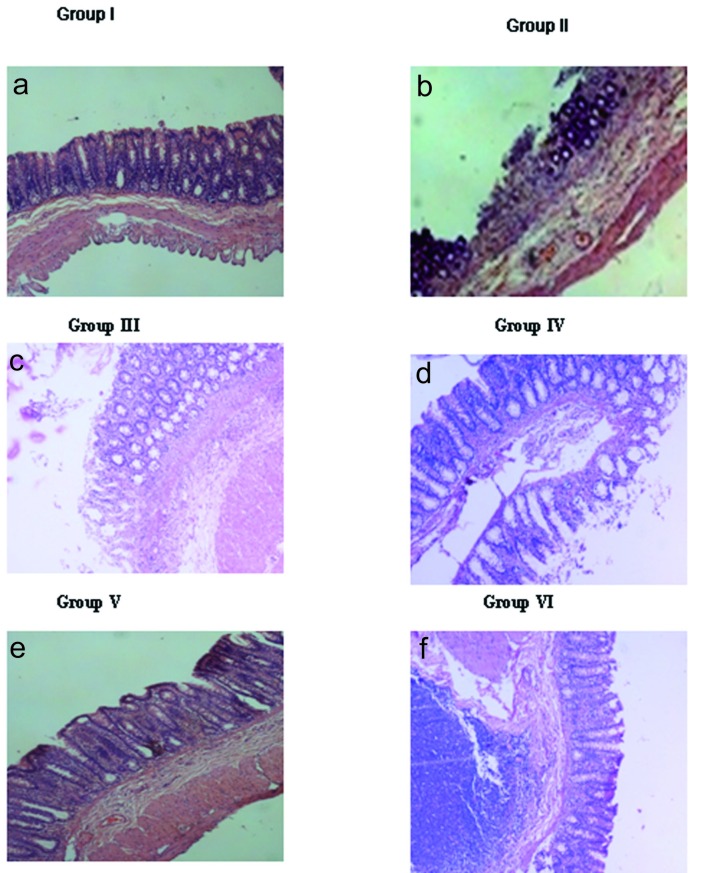
Histopathology. a) Normal mucosa seen in Group I animals; b) Ulceration (arrow) with inflammatory exudates and denudation of mucosal glands induced with only naproxen in Group II; c) Denudation of epithelial lining (arrow) with inflammatory cells in mucosa and submucosal layer in Group III animals (7 days); d) Mucosa of rhEGF treated animals, Group IV (7 days); e) Incomplete regeneration of mucosa (arrow) seen along with lymphoid hyperplasia in Group V animals (14 days); f) Complete regeneration of mucosa seen in the rhEGF treated animals, Group VI (14 days). Magnification is 100 x for all the images. The data represented are from three independent identical experiments.

The Group I animals demonstrated normal morphology with mucosal integrity of the gastro-antral region. However, the Group II animals showed gastric antral ulcers with denudation of mucosal glands and inflammatory exudates. Similarly, Group III and Group V animals showed more of denudation of epithelial lining with inflammatory cells in submucosal layer similar to that of Group II animals. However, administration of rhEGF to the naproxen treated animals ameliorated the pathological changes of gastric mucosa partially with some inflammatory changes still persisting in the gastric mucosa at 7 days (Group IV), with a total amelioration seen by 14 days (Group VI). The cytoarchitecture of the gastric antral region of the Group VI animals was comparable to Group I (Fig. 1).

### Measurement of serum TBARS

**Figure 2:**
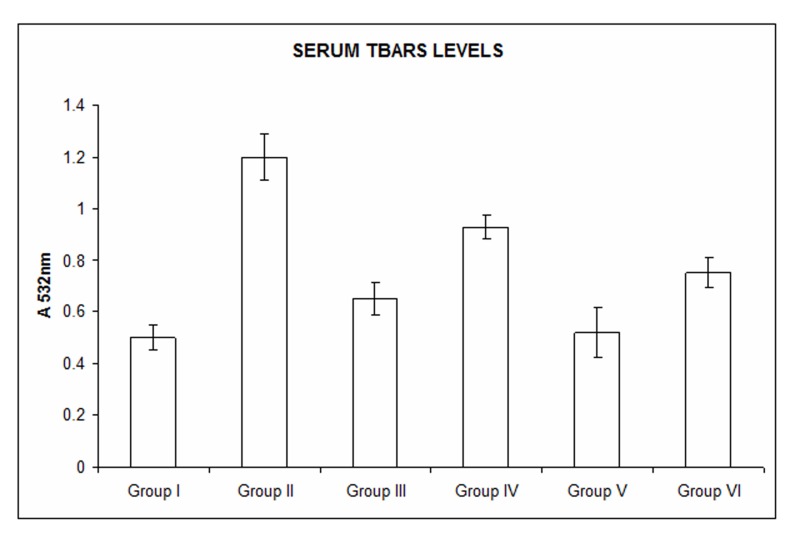
Measurement of serum TBARS. Serum TBARS levels were significantly increased with naproxen treatment in Group II, Group III and Group V animals. TBARS levels were decreased with rhEGF treatment in Group IV and Group VI animals which were comparable to the Group I (controls). Data are the means ± SE for three independent experiments. (P < 0.05 compared to Group I, Group IV and Group VI by one way ANOVA).

The TBARS levels in the serum were significantly increased in the naproxen treated animals (Group II, Group III and Group V). With rhEGF treatment, there was an appreciable decrease in the serum TBARS content which was comparable to that of the control animals. The significance was at P value of less than 0.05 (Fig. 2).

### Immunolocalization of Cox-2

**Figure 3:**
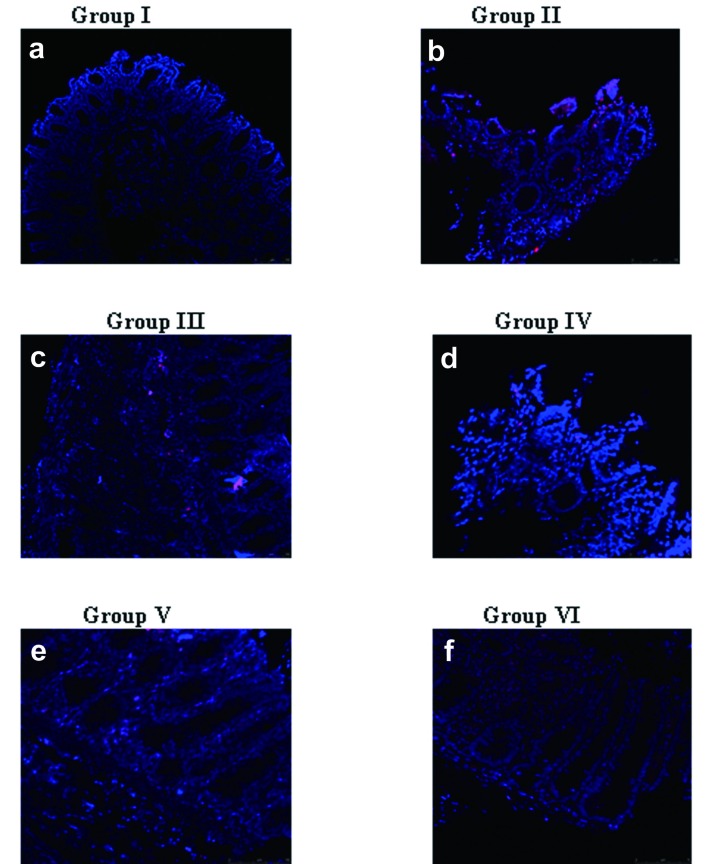
Immunolocalization of Cox-2. Cox-2 localization was weak in: a) Group I animals; d) rhEGF treated Group IV (7days); f) rhEGF treated Group VI (14days). However, a predominant localization was seen in naproxen treated animals such as: b) Group II; c) Group III; e) Group V. The data represented are from three independent identical experiments and the images have been captured with Nikon Inverted fluorescence microscope TE 2000-S series.

Treatment with naproxen (Group II, Group III and Group V) demonstrated Cox-2 localization seen predominantly in the ulcerated region of the gastric antral tissue. Interestingly, with rhEGF treatment, the Cox-2 localization was reduced to a larger extent (Group IV) and was almost absent and comparable to that of the controls by 14 days of treatment (Group VI) (Fig. 3). The relative fluorescent unit (RFU) of the Cox-2 immunolocalization in control and the experimental groups was calculated (data not shown).

### Semi-quantitative RT-PCR

**Figure 4:**
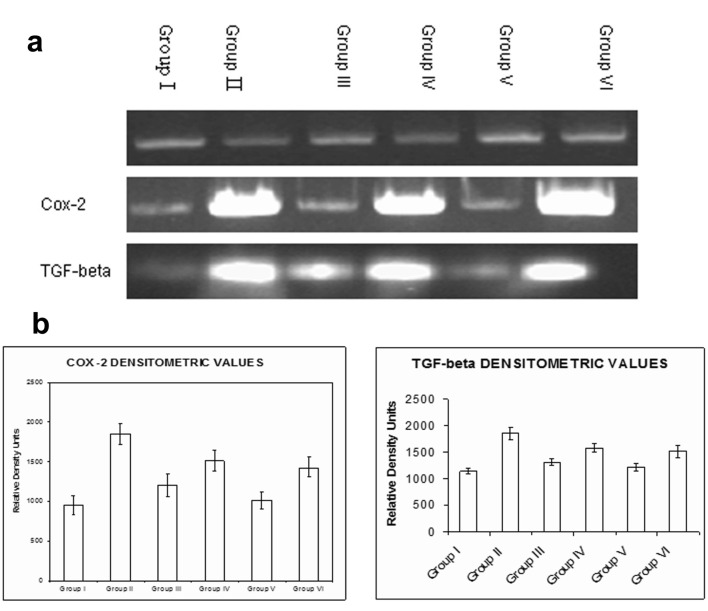
Semi-quantitative RT-PCR. a) An increase in the expression of both Cox-2 and TGF-b were seen with naproxen treatment in Group II, Group III and Group V animals. Administration of rhEGF decreased the expression of Cox-2 and TGF-b in Group IV and Group VI animals which were similar to the Group I (controls); b) The densitometry analysis of the RT-PCR data for Cox-2 and TGF-b. Data are the means ± SE for three independent experiments. (P < 0.05 compared to Group I, Group IV and Group VI by one way ANOVA).

Treatment with naproxen (Group II, Group III and Group V) showed an upregulation in the expression of Cox-2 and TGF-β. Administration of rhEGF to the naproxen treated animals decreased the expression of both the genes Cox-2 and TGF-β which was more significant in Group VI animals similar to the Group I animals. However, the reduction in the expression of the Cox-2 and TGF-β was of lesser magnitude in the 7 days of rhEGF treatment (Group IV). β actin was used as the house-keeping gene in each assay (Fig. 4a). The quantitative changes in the expression for both Cox-2 and TGF-β have been indicated by densitometric scanning (Fig. 4b).

## Discussion

The present study has been carried out to explore the healing effects of rhEGF in the naproxen induced gastric antral ulcers treated either for 7 days or for 14 days. Earlier studies have reported the efficacy of EGF in negating the detrimental effects of the gastric mucosal ulcers both in acute and chronic conditions treated with ethanol [11]. However, these studies are limited at several levels in understanding the cytoprotection and repair mechanism(s) underlying the healing process [18]. The rhEGF which has been developed indigenously by BBIL India [12] have proven to be effective in the healing of diabetic foot ulcers, skin burns and wound healing when tested in the human subjects [13]. Literature survey shows that there have been no studies undertaken to implicate the beneficial effects of rhEGF in the management of gastric antral ulcers induced with NSAID keeping in view of the fact that NSAIDs have been frequently used as anti-inflammatory drugs for arthritic patients and that gastric ulceration is widely prevalent especially in the antral region [9]. Our present study forms a novel approach to investigate the pathology, biochemical, cellular and molecular changes associated in the naproxen induced gastric antral ulcers and their healing with rhEGF offering a suitable model system to the human situation.

EGF is mitogenic and its proliferative effect is believed to contribute to the maintenance of mucosal integrity within the gastrointestinal tract [18]. In our present study, the lag time from ulcer induction to complete gastric healing with rhEGF treatment was 14 days and the benefits of such agents would include a higher therapeutic index and lower toxicity than conventional therapies [13]. Further, exogenous EGF administration given parenterally or orally, stimulated the growth of gastric/duodenal mucosa and was shown to be effective in restoration of the damage induced with acetic acid/ethanol treatment [19]. However, there are other reports to show that oral administration of EGF at 30 and 100 µg/kg bodyweight for 2 weeks had no effect on the ulcer healing in Donryu rats with gastric ulcers induced by submucosal injection of acetic acid into the antral region. These variations probably could be attributed to the different strains of animals, method of ulcer induction, severity of ulcer formation used and duration of ulcer treatment [20].

We examined the histopathology of the gastric antral tissues by Hematoxylin and Eosin of the control and the experimental Groups. The results undoubtedly demonstrated the healing rendered by rhEGF as evidenced by the normalization of cytoarchitecture of the gastric mucosa by 14 days with some inflammatory changes still persisting in the early phase of the treatment (7 days) (Fig. 1). Studies have shown that the ulcerative lesions of gastro-antral tract are one of the major side effects of NSAID(s) and limit their usage on long-term basis [9]. Supporting these studies, our data also substantiate that with naproxen treatment, gastric antral lesions underlying the ulcerative changes are appreciable. This has been manifested with denudation of mucosal glands and inflammatory exudates seen in Group II, Group III and Group V animals. Naproxen is a non-corticosteroid drug with anti-inflammatory and antipyretic effects causing erosions, antral ulceration and petechial bleeding in the mucosa of stomach [5, 6]. It has been demonstrated that the production of oxygen free radicals and lipid peroxidation plays a crucial role in the development of the gastric antral ulcers induced by NSAIDs [7, 8]. In similar lines, we have also demonstrated an increase in the lipid peroxides when treated with naproxen (NSAIDs), which was negated by the beneficial effects of oral administration of rhEGF. Further, the beneficial effects to overcome the naproxen induced rise in lipid peroxide seen specifically in gastric antral ulcers with astaxanthin treatment are of therapeutic efficacy [9].

The role of Cox-2 in ulcer and healing process has been well documented [21]. However, there are conflicting reports in literature attributed to the differences in the specificity of the antibodies used or the species variations [22, 23], where the Cox-2 expression has been demonstrated even in the normal gastric mucosa. However, our present data are in line with the studies reported by Lajoie et al [24] which correlates for the increased Cox-2 localization attributed to the mucosal lesions seen in the gastric antral ulcers induced with naproxen [25-27]. Interestingly, with the total amelioration of the gastric mucosa seen by 14 days (Group VI), vis-a-vis, the Cox-2 localization was minimal or non detectable and comparable to the normal gastric mucosa of the controls. This suggests the process of healing and repair induced with rhEGF treatment.

Ulcer healing is a complex process which involves cascade of events and interactions regulated by the expression of several factors. Studies by Satoru [28] have reported an increase in the expression of TGF-β in tissue repair and remodeling process akin to our present data demonstrating the increased TGF-β and Cox-2 expression in naproxen induced gastric lesions. The healing of the gastric antral ulcers which was almost evident by 14 days of rhEGF treatment correlated well with the decreased TGF-β and Cox-2 mRNA expression. Parallelly, Tominga et al [29] have also correlated the TGF-β mRNA expression to the wound and healing process. In addition, there are some recent studies showing an induction in the HGF expression synergistic with Cox-2 expression in the gastric ulcer healing process [30, 31]. Further, the use of specific inhibitors of Cox-1 and Cox-2 such as NS-398 and indomethacin have shown to impair the healing of ulcer which suggests for an important role of endogenous growth factors in mucosal repair [4].

In conclusion, the present study forms the basis to be reported for the first time the efficacy of rhEGF in overcoming/mitigating the gastro-antral ulcer induced with naproxen. In this experimental model, we have attempted to correlate the ulcer healing process by approaches such as histopathology, lipid peroxide levels, Cox-2 immunolocalization and expression of the tissue healing markers such as Cox-2 and TGF-β genes. We believe that exploring the potential of rhEGF to understand the mechanism(s) (biochemical, cellular and molecular) of healing carried out in the model system will help in advancing our knowledge towards the management of antral gastric ulcers.
